# Neuraminidase-specific antibodies drive differential cross-protection between contemporary FLUBV lineages

**DOI:** 10.1126/sciadv.adu3344

**Published:** 2025-03-28

**Authors:** Caroline K. Page, Justin D. Shepard, Sean D. Ray, James A. Ferguson, Alesandra J. Rodriguez, Julianna Han, Joel C. Jacob, Dawne K Rowe-Haas, Jasmine Y. Akinpelu, Lilach M. Friedman, Tomer Hertz, Andrew B. Ward, Stephen M. Tompkins

**Affiliations:** ^1^ Center for Vaccines and Immunology, University of Georgia, Athens, GA 30602, USA.; ^2^ Center for Influenza Disease and Emergence Response (CIDER), University of Georgia, Athens, GA 30602, USA.; ^3^ Department of Infectious Diseases, University of Georgia, Athens, GA 30602, USA.; ^4^ Department of Integrative Structural and Computational Biology, The Scripps Research Institute, La Jolla, CA 92037, USA.; ^5^ The Shraga Segal Department of Microbiology and Immunology, Ben-Gurion University of the Negev, Beer-Sheva 8410501, Israel.; ^6^ National Institute of Biotechnology in the Negev, Ben-Gurion University of the Negev, Beer-Sheva 8410501, Israel.; ^7^ Vaccine and Infectious Disease Division, Fred Hutch Cancer Research Center, Seattle, WA 98109, USA.

## Abstract

The two influenza B virus (FLUBV) lineages have continuously diverged from each other since the 1980s, with recent (post-2015) viruses exhibiting accelerated evolutionary rates. Emerging data from human studies and epidemiological models suggest that increased divergence in contemporary viruses may drive differential cross-protection, where infection with Yamagata lineage viruses provides limited immunity against Victoria lineage viruses. Here, we developed animal models to investigate the mechanisms behind asymmetric cross-protection between contemporary FLUBV lineages. Our results show that contemporary Victoria immunity provides robust cross-protection against the Yamagata lineage, whereas Yamagata immunity offers limited protection against the Victoria lineage. This differential cross-protection is driven by Victoria-elicited neuraminidase (NA)–specific antibodies, which show cross-lineage reactivity, unlike those from Yamagata infections. These findings identify a phenomenon in contemporary FLUBV that may help explain the recent disappearance of the Yamagata lineage from circulation, highlighting the crucial role of targeting NA in vaccination strategies to enhance cross-lineage FLUBV protection.

## Teaser

FLUBV Victoria infection elicits NA-specific antibodies that provide cross-lineage protection, possibly driving Yamagata decline.

## INTRODUCTION

The divergent evolutionary trajectories of the two influenza B virus (FLUBV) lineages, Victoria and Yamagata, present unique opportunities in understanding immune responses and cross-protection between contemporary viruses. FLUBV, first identified in the 1940s (*1*), evolved into two distinct lineages in the late 1970s and early 1980s (*2*–*4*). Despite occasional reassortment between them, the lineages are primarily defined by differences in their hemagglutinin (HA) sequences (*3*, *5*). Both undergo insertions and deletions in the immunodominant glycoproteins, HA and neuraminidase (NA), leading to antigenic drift and enabling viral escape from preexisting immunity (*3*, *6*). This process is particularly evident in post-2015 FLUBV isolates, which exhibited increased endemic activity, accelerated evolution, and multiple selective sweeps, further diversifying each lineage into distinct clades and subclades (*7*, *8*). A notable example is the emergence of the Victoria subclade V1A.3 between 2018 and 2019, which dominated previous subclades and contributed to the unusual 2019–2020 influenza season, where FLUBV infections peaked earlier than influenza A virus (FLUAV) infections (*9*).

Modeling and retrospective studies have explored immunity to influenza viruses, focusing on FLUAV and, to a lesser extent, FLUBV (*10*–*13*). In humans, homotypic or intralineage protection is observed against reinfection with FLUAV and FLUBV viruses, respectively. Immunity gained from H1N1, H3N2, and Victoria lineage viruses can protect against reinfection with the same virus or lineage in subsequent seasons. However, Yamagata lineage viruses provide limited protection within the lineage, in addition to minimal cross-lineage protection (*11*). For instance, infection with Yamagata in one season offers only minimal protection against a Victoria infection in the next (*10*). Limited protection from reinfection after exposure to Yamagata viruses observed in humans could potentially justify higher season-to-season reinfections associated with this lineage (*10*, *14*). Furthermore, the potential extinction of the Yamagata lineage following the COVID-19 pandemic has prompted the shift from quadrivalent to trivalent influenza vaccines, raising concerns about a Yamagata resurgence and highlighting gaps in our understanding of cross-lineage immunity (*15*, *16*).

The humoral immune response plays an integral role in influenza protection by targeting the two major viral surface proteins: HA and NA (*17*–*19*). Most antibodies elicited by influenza infections or vaccinations target HA, a protein crucial for viral entry into host cells (*20*, *21*). Although HA-specific antibodies, particularly those targeting its immunodominant head domain, play a key role in neutralization, their cross-reactivity across strains and lineages is limited due to the high mutation rates of the HA globular head (*7*). Recent attention has turned to NA-specific antibodies, which have shown promise in cross-protective immunity (*22*–*28*). Unlike HA, NA undergoes less antigenic variation, and NA-specific antibodies can inhibit viral egress, curtailing viral replication and reducing disease severity. However, despite their potential, the role of NA antibodies in mediating cross-protection between the Victoria and Yamagata lineages remains poorly understood, and contemporary virus mutations may affect antibody efficacy.

Although human studies have revealed interesting patterns in FLUBV infection and reinfection, experimental animal models that mimic these dynamics are lacking, and the mechanisms underlying these differences remain unclear. Here, we establish two animal models to replicate preliminary human observations and demonstrate that NA-specific antibodies are key mediators of cross-protection from contemporary Victoria to Yamagata viruses. These findings offer important insights into the asymmetric immunity between FLUBV lineages and how viral evolution influences immune responses.

## RESULTS

### Influenza preimmunity affects protection against a cross-lineage influenza B infection

To assess the immune response to contemporary FLUBV lineages, B/Washington/02/2019 (B/WA) was chosen to represent the Victoria lineage and B/Oklahoma/10/2018 (B/OK) was selected to represent the Yamagata lineage. B/Washington/02/2019 was included in the 2020–2021 quadrivalent influenza vaccine and belongs to the recently emerged subclade, V1A.3, of the Victoria lineage (*29*). B/Oklahoma/10/2018 (clade 3A), which is antigenically similar to the Yamagata vaccine isolate, was circulating in the United States just prior to the onset of the COVID-19 pandemic. To replicate the limited cross-protection observed in humans following contemporary Yamagata virus infections, FLUAV preimmune ferrets, previously exposed to A/California/07/2009 (H1N1), along with naïve ferrets were intranasally inoculated with either B/Washington/02/2019 (Victoria) or B/Oklahoma/10/2018 (Yamagata) at a dose of 10^6^ plaque-forming units (PFU). No clinical signs, such as weight loss or temperature changes, were observed in any group (fig. S1, A to D), and FLUBV replication was comparable in the upper respiratory tract of H1N1-preimmune and naïve ferrets for both lineages. These findings suggest that H1N1 prior immunity does not affect initial FLUBV infection (fig. S1, E and F).

**Fig. 1.**
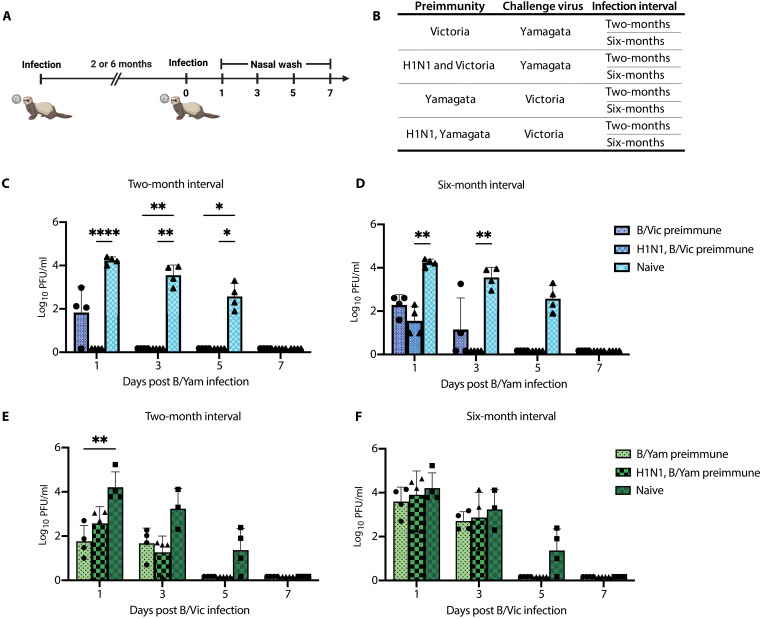
Influenza preimmunity contributes to the duration of cross-protection elicited between Victoria and Yamagata lineage viruses. (**A** and **B**) Experimental design, timeline, and immune status for establishing a ferret FLUBV cross-protection model. Naïve or A/California/07/2009 (H1N1) preimmune ferrets were intranasally inoculated with 10^6^ PFU of either B/Washington/02/2019 (B/Vic) or B/Oklahoma/10/2018 (B/Yam). Following the initial FLUBV infection, animals were rested for either 2 or 6 months and then infected with the opposing lineage influenza B virus. (**C** and **D**) Viral titers in nasal wash samples collected days 1, 3, 5, and 7 postsecondary FLUBV infection in ferrets with B/Vic preimmunity, challenged with B/Yam. (**E** and **F**) Viral titers from nasal wash samples in ferrets with B/Yam preimmunity, challenged with B/Vic after a 2- or- 6-month rest period. Viral titers from naïve ferrets in (C) and (D) and (E) and (F) are repeated for graphical clarity. Statistical significance was determined by a two-way ANOVA with Geisser-Greenhouse correction, with *P* values indicated as follows: *P < 0.05, **P < 0.01, and ****P < 0.0001. Data points represent individual animals, and bars represent means ± SD.

To assess differences in duration of cross-protection following initial FLUBV infection, the ferrets were rested for either 2 or 6 months prior to being challenged with the opposing influenza B lineage virus (Fig. 1, A and B). Two months following a Victoria exposure, H1N1-preimmune ferrets with Victoria immunity were fully protected against Yamagata challenge as they had no detectable viral replication in the upper respiratory tract postinfection (Fig. 1C). Among Victoria-immunized animals without H1N1 preimmunity, one showed complete protection, while the other three trended toward lower viral replication compared to the naive control on day 1 postinfection, with full viral clearance by day 3. (Fig. 1C). At 6 months post-Victoria infection, H1N1-preimmune ferrets exhibited significantly lower viral replication on day 1 following Yamagata infection compared to naïve controls, whereas Victoria-only preimmune animals showed a trend toward reduced viral replication (Fig. 1D). However, two of the Victoria-only animals still exhibited viral shedding on day 3 postinfection (Fig. 1D).

Conversely, Yamagata preimmune ferrets exhibited a reduction in viral shedding only on day 1 post-Victoria infection at the 2-month infection interval (Fig. 1E). At the 6-month interval, viral shedding in the Yamagata preimmune group was equivalent to naïve control-infected ferrets on days 1 and 3 (Fig. 1F). Yamagata preimmune ferrets with H1N1-preimmunity displayed no reduction in viral titers at the 2- or 6-month infection intervals (Fig. 1, E and F). The virus was cleared by day 5 in all Yamagata preimmune animals, whereas three of four naïve animals continued shedding virus at this time point (Fig. 1F).

These data suggest that contemporary FLUBV isolates elicit differential cross-protection, with the Victoria lineage conferring stronger cross-lineage protection than Yamagata. Furthermore, H1N1 preimmunity combined with Victoria immunity offers enhanced protection compared to Victoria immunity alone but does not improve protection when coupled with Yamagata immunity.

### Differential cross-protection is a phenomenon for contemporary viruses

**Fig. 2.**
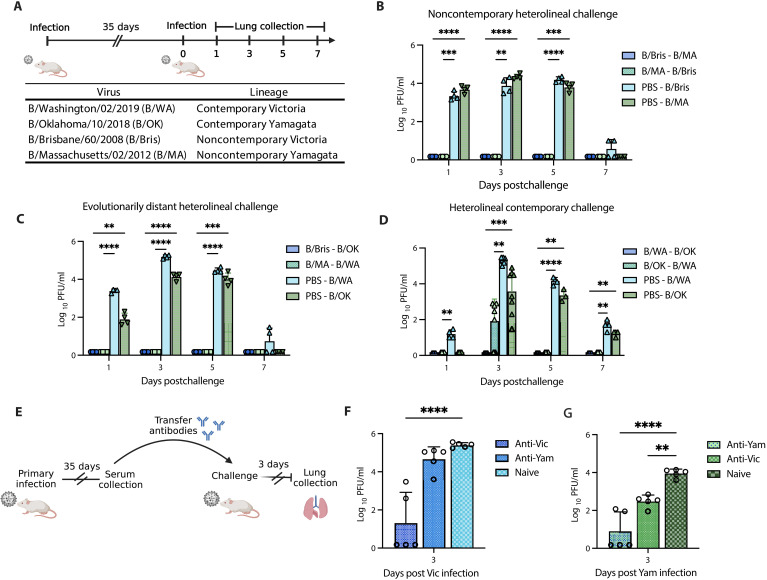
Serum antibodies mediate differential protection against influenza B with contemporary viruses. (**A**) Experimental design, timeline, and viruses used for establishing the FLUBV cross-protection model. Mice were intranasally infected with 10^3^ PFU of either B/WA, B/OK, B/Bris, or B/MA, rested for 35 days, and then reinfected with 10^4^ PFU of an opposing lineage virus. Lung samples were collected on days 1, 3, 5, and 7 postsecondary infection to assess viral replication. (**B**) Viral replication in the lungs of mice initially infected with noncontemporary B/Bris or B/MA and then reinfected 35 days later with cross-lineage noncontemporary B/MA or B/Bris, respectively. (**C**) Viral replication in the lungs of mice initially infected with noncontemporary B/Bris or B/MA and then reinfected 35 days later with cross-lineage contemporary B/OK or B/WA, respectively. (**D**) Viral replication in the lungs of mice initially infected with contemporary B/WA or B/OK and then reinfected 35 days later with cross-lineage contemporary B/OK or B/WA, respectively. The day 3 data represent two independent experiments. (**E**) Experimental design for the passive transfer experiment. Mice were intranasally inoculated with 10^3^ PFU of B/WA or B/OK. Serum was collected 35 days postinfection and transferred to naïve recipient mice, which were then challenged with 10^3^ PFU of the cross-lineage virus, B/OK or B/WA, respectively. Lungs were collected from challenged mice on day 3 postinfection. (**F**) Viral replication in the lungs of mice passively transferred anti-Vic or anti-Yam antibodies and challenged with B/WA (Vic). (**G**) Viral replication in the lungs of mice passively transferred anti-Vic or anti-Yam antibodies and challenged with B/OK (Yam). Statistical significance was determined using a two-way [(B) to (D)] or one-way [(F) to (G)] ANOVA, with **P < 0.01, ***P < 0.001, and ****P < 0.0001.

Considering the notably divergent evolutionary trajectories FLUBV lineages have undergone in recent years, we sought to investigate whether the differences in cross-protection between Victoria and Yamagata viruses is restricted to contemporary viruses belonging to recently emerged FLUBV clades or whether this phenomenon also spans evolutionarily older viruses as well. Mice were intranasally infected with a sublethal dose of 10^3^ PFU of either a contemporary or noncontemporary Victoria or Yamagata virus (Fig. 2A). None of the infections resulted in significant weight loss (fig. S2, A and B), but viral replication occurred at similarly high titers in the lungs of the mice, except on days 1 and 2 postinfection, when the contemporary Victoria virus exhibited higher replication than the contemporary Yamagata virus (fig. S2, C and D).

Thirty-five days postinfection, after clearance of the acute infection and the establishment of immune memory, the animals were reinfected with a contemporary or noncontemporary cross-lineage challenge virus, and lungs were collected to assess viral replication (Fig. 2A). First, we explored whether a noncontemporary infection could provide cross-lineage protection, which was not observed with contemporary viruses in ferrets. Mice were primarily infected with B/Brisbane/60/2008 (B/Bris) or B/Massachusetts/02/2012 (B/MA) and challenged with the opposing lineage viruses [i.e., B/Bris (Victoria) primed mice were challenged with B/MA (Yamagata) and B/MA primed mice were challenged with B/Bris]. We observed complete cross-lineage protection with noncontemporary viruses (Fig. 2B). Next, we sought to understand whether the noncontemporary primary infection could also provide protection from the viruses belonging to evolutionarily distant “contemporary” clades. We show animals primarily infected with B/Bris or B/MA were completely protected from a challenge with contemporary B/OK or B/WA, respectively (Fig. 2C). In contrast, contemporary Yamagata (B/OK) infection provided incomplete protection from a contemporary Victoria (B/WA) challenge, with five of eight mice showing breakthrough infection on day 3 postchallenge (Fig. 2D).

These findings in the mouse model mirrored the results observed in ferrets, strengthening the evidence that contemporary Yamagata strains elicit less robust cross-lineage protection compared to Victoria strains. In addition, these data complement our ferret data, demonstrating that this phenomenon is specific to contemporary viruses, with noncontemporary strains providing broader cross-protection that spans FLUBV evolution.

### Serum antibodies mediate differential protection elicited from contemporary influenza B virus infections

To investigate the mechanism underlying this differential protection, we assessed the role of serum antibodies in mediating the differences observed in cross-protection. Mice were passively transferred serum conferred from either a contemporary Victoria or Yamagata infection and subsequently challenged intranasally with 10^3^ PFU of a cross-lineage contemporary virus (Fig. 2E). Serum antibodies elicited by Victoria infection significantly reduced viral replication following homologous Victoria challenge (Fig. 2F) and cross-lineage Yamagata challenge (Fig. 2G). Conversely, Yamagata-elicited antibodies significantly reduced viral replication during homologous Yamagata challenge (Fig. 2G) but failed to protect against cross-lineage Victoria challenge (Fig. 2F).

Together, these data suggest that serum antibodies alone are capable of mediating the differential protection observed in contemporary influenza B virus infections. Furthermore, the findings highlight the superior cross-lineage protection conferred by Victoria-elicited antibodies compared to those elicited by Yamagata infections.

### HA-specific influenza B antibodies elicit homologous protection

The defined divergence between the FLUBV lineages is based on sequence homology of the HA protein. Therefore, observations of differential immunity in our animal model prompted us to hypothesize that HA-specific antibodies alone are sufficient in driving differential cross-protection. We investigated whether broadly reactive HA antibodies are responsible for the differential protection observed between contemporary and noncontemporary FLUBV infections. To evaluate cross-reactivity, we performed hemagglutination inhibition (HI) assays using serum from both contemporary and noncontemporary infections in mice. Noncontemporary viruses elicited broader HI titers against both homologous (B/MA) and heterologous (B/Bris) antigens compared to contemporary viruses, suggesting that the broader protection observed in noncontemporary infections may be driven by HA-specific antibodies (fig. S2E). In contrast, HI titers from contemporary viruses were restricted to homologous antigens.

**Fig. 3.**
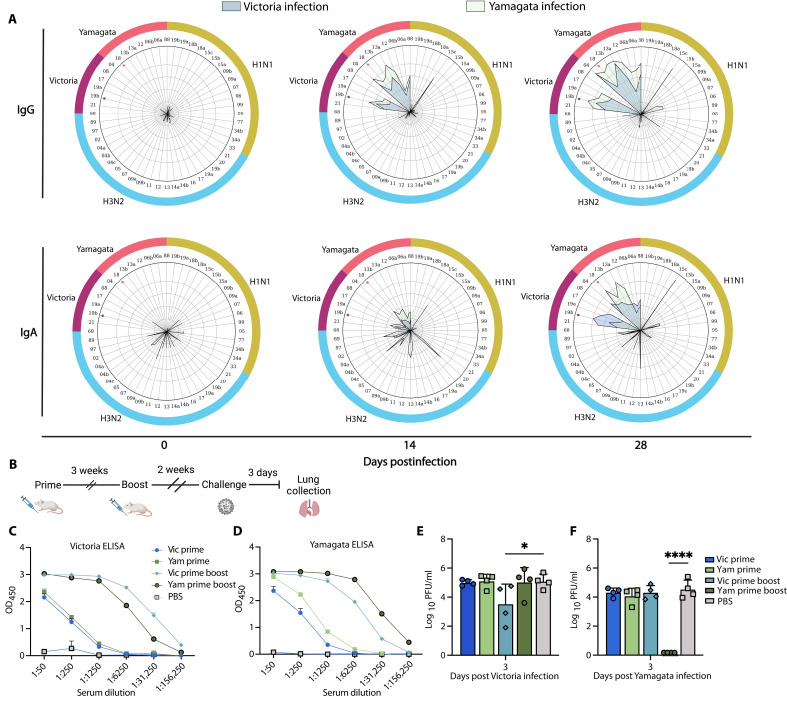
HA-specific antibodies provide homologous protection against an FLUBV challenge. (**A**) Spider plots displaying average normalized IgG (top) and IgA (bottom) serum responses in Victoria- and Yamagata-infected mice for various influenza A and B antigens: Yamagata (pink), Victoria (purple), H1N1 (yellow), and H3N2 (light blue). Each vertex represents the normalized binding of IgG/IgA to a single influenza antigen. Anti-WA (Victoria) infection serum and anti-OK (Yamagata) infection serum groups are denoted by blue and green lines, respectively. The outer circle numbers indicate which antigen the response is directed at (table S1), whereas the pink and purple dots inside the circle denote HA antigens of the infection strains Victoria (B/WA) and B/Yamagata (B/OK), respectively. (**B**) Timeline for rHA vaccination in mice with either B/WA HA (Victoria) or B/OK HA (Yamagata). (**C** and **D**) ELISA of serum collected after prime or prime-boost vaccination, tested against rHA from homologous or cross-lineage antigens. OD, optical density. (**E** and **F**) Lung viral titers on day 3 postinfection with contemporary FLUBV (B/WA, Victoria or B/OK, Yamagata) in rHA-vaccinated mice. Statistical significance was determined using a one-way ANOVA, with *P < 0.05 and ****P < 0.0001.

To further define the breadth of the HA-elicited immunoglobulin G (IgG) and IgA serum responses following infection with contemporary Victoria and Yamagata viruses, we used an antigen microarray spotted with multiple recombinant HA (rHA) proteins from FLUAV and FLUBV antigens (table S1) (*30*). Serum from mice infected with either the contemporary Victoria or contemporary Yamagata was collected on days 0, 14, and 28 postinfection for analysis. The IgG and IgA responses elicited from both infections show cross-reactivity to a diverse set of antigens spanning lineage and evolutionary clade (Fig. 3A).

To assess the protective capacity of these cross-reactive HA antibodies elicited from infection, naive mice were vaccinated with rHA from contemporary Victoria and Yamagata viruses in a prime-boost regimen, followed by cross-lineage challenge (Fig. 3B). Although cross-reactive IgG antibodies were elicited after both the prime and boost (Fig. 3, C and D), only homologous protection was observed upon challenge, with no heterolineal cross-protection elicited by rHA vaccination (Fig. 3, E and F). Together, these findings suggest that, although cross-reactive anti-HA IgG and IgA antibodies are elicited by FLUBV infection, functionally, they are sufficient only for homologous protection and do not mediate the differential cross-protection observed between the Victoria and Yamagata lineages.

### Yamagata-elicited NA antibodies drive differential cross-protection

**Fig. 4.**
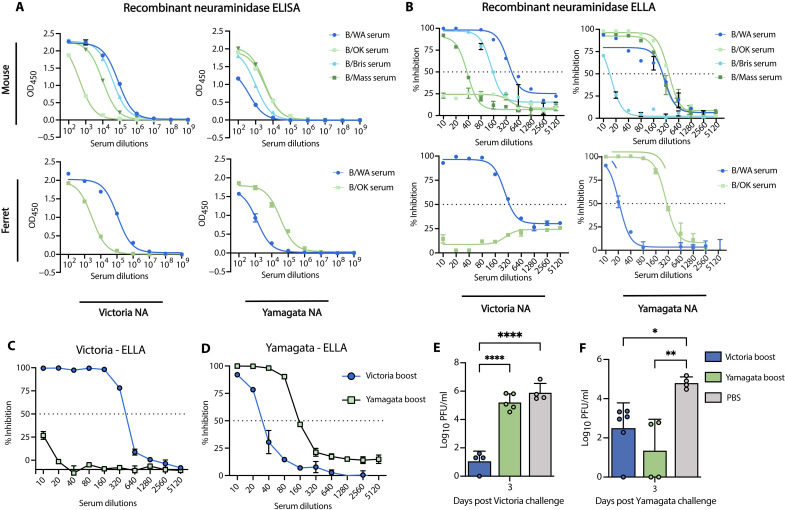
Functional NA-specific antibody responses drive differential cross-protection against contemporary FLUBVs. (**A**) ELISA following FLUBV infection with either B/Washington/02/2019 (B/WA), B/Oklahoma/10/2018 (B/OK), B/Brisbane/60/2008 (B/Bris), or B/Massachusetts/02/2012 (B/MA) in mice or ferrets, tested against rNA from Victoria (B/WA) or Yamagata (B/OK) lineages. (**B**) ELLA following FLUBV infection with B/WA, B/OK, B/Bris, or B/MA in mice and ferrets. Dashed line indicated the 50% inhibition cutoff. (**C** and **D**) ELLA results using serum from mice postboost with rNA vaccination. (**E** and **F**) Lung viral titers on day 3 postinfection with contemporary FLUBV (B/WA, Victoria or B/OK, Yamagata) in rNA-vaccinated mice. Statistical significance was determined using a one-way ANOVA, with *P < 0.05, **P < 0.01, and ****P < 0.0001.

After demonstrating that HA-specific antibodies do not mediate differential cross-protection between contemporary influenza B virus lineages, we next explored whether NA-specific antibodies contributed to cross-lineage protection. Serum collected 4 weeks after infection with contemporary FLUBVs (B/WA and B/OK) in both mice and ferrets, and noncontemporary FLUBVs (B/Mass and B/Bris) in mice only, showed cross-reactivity with both homolineal and heterolineal antigens, as measured by enzyme-linked immunosorbent assay (ELISA) using recombinant NA (rNA) (Fig. 4A and fig. S4, A and B). However, after determining the functional ability of these NA directed antibodies to inhibit the activity of NA with enzyme-linked lectin assay (ELLAs), we found that contemporary Yamagata-elicited anti-NA antibodies were deficient at inhibiting contemporary Victoria NA activity in both mice and ferrets. In contrast, the noncontemporary Yamagata and both contemporary and noncontemporary Victoria lineage viruses elicited cross-reactive NA-specific serum antibodies that inhibited NA activity from both lineages (Fig. 4B and figs. S2F and S4, C and D). These data suggest that functional cross-reactive anti-NA serum antibody responses are driving the differential protection seen in both mice and ferrets with contemporary FLUBVs.

To confirm that the phenomenon is driven by cross-reactive functional anti-NA antibodies, we vaccinated mice in a prime and boost regimen with the rNA of a contemporary Victoria (B/WA) and Yamagata (B/OK) virus. Two weeks following the boost, serum was collected and the mice were challenged with either the homologous or cross-lineage virus. Three days following challenge, lungs were collected to assess viral replication. ELLAs revealed that serum elicited from rNA immunization displayed asymmetric functional differences also seen by infection whereby Yamagata immunity does not have cross-functional antibodies that inhibit NA activity of the Victoria NA (Fig. 4C). However, Victoria rNA immunization elicited cross-functional anti-NA antibodies (Fig. 4D). We also observed that rNA vaccination provides homologous protection by reducing viral replication compared to naive infected controls (Fig. 4, E and F) similar to the HA vaccination (Fig. 3, F and G). However, in contrast to rHA vaccination, our NA vaccination shows cross-lineage reduction in viral replication occurs in mice immunized with the Victoria rNA but not with the Yamagata rNA (Fig. 4, E and F). Furthermore, vaccine-elicited anti-NA antibodies elicited from both Yamagata and Victoria rNA vaccination were equally able to reduce viral replication from a Yamagata infection (Fig. 4F).

Together, these findings demonstrate that functional NA-specific antibodies mediate the differential protection observed between influenza B lineages. Serum analysis from infection and vaccination experiments orthogonally show deficiencies in Yamagata-elicited anti-NA antibodies, whereas rNA vaccination demonstrates that anti-NA–elicited immunity can recapitulate differential infection dynamics observed in both mice and ferrets.

### Structural analysis reveals the absence of cross-reactive antibodies from Yamagata infection

**Fig. 5.**
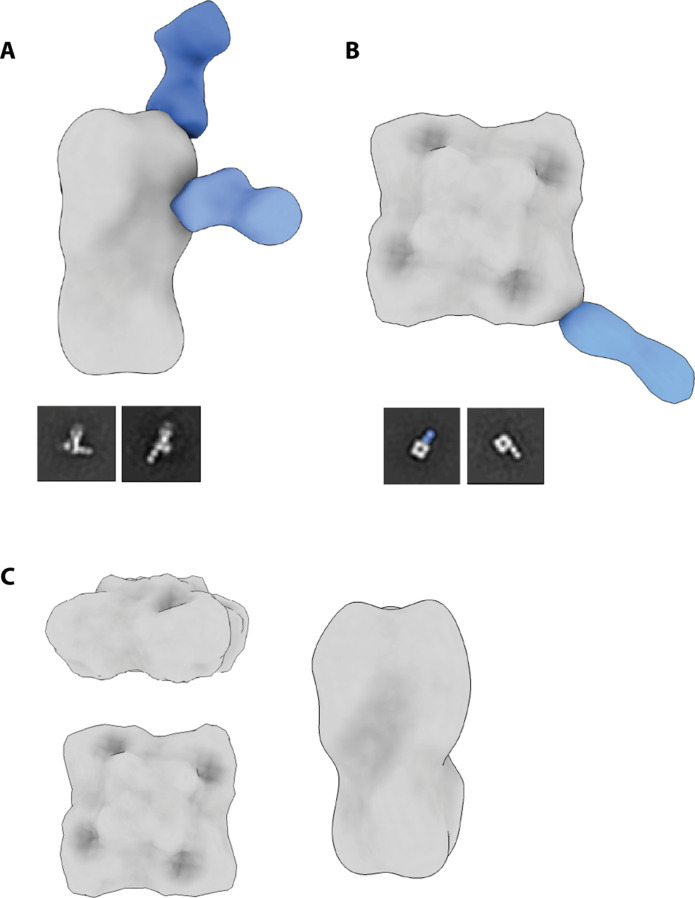
Negative-stain electron microscopy reconstructions of Victoria ferret pAbs with representative 2D classes. pAb complex assemblies were generated by segmenting and resampling densities corresponding to each Fab and then mapped onto an HA trimer or NA tetramer. Complete 3D classes are shown in the Supplementary Materials. (**A**) Recombinant B/Washington/02/2019 (B/WA; Victoria) HA with B/WA ferret serum at day 28 postinfection. (**B**) Recombinant B/WA NA with B/WA ferret serum at day 28 postinfection. A putative NA interface pAb is highlighted in 2D as insufficient particles were available for a 3D reconstruction. (**C**) Recombinant B/WA HA and NA complexed with B/Oklahoma/10/2018 (B/OK; Yamagata) ferret serum at day 28 postinfection. No pAb responses observed.

To visualize the polyclonal antibody (pAb) response from FLUBV, we used electron microscopy polyclonal epitope mapping (EMPEM). We analyzed sera from ferrets infected with a Victoria (B/Washington/02/2019) or a Yamagata (B/Oklahoma/10/2018) strain on day 28, in complex with recombinantly expressed Victoria HA and NA proteins. EMPEM revealed two HA pAbs from the B/Washington/02/2019 infection. One pAb bound to the side of the HA head of the Victoria HA protein, another binding to the top of the HA head, both corresponding to major antigenic sites (Fig. 5A) (*31*). In particular, the top pAb appears to bind to the 150-loop antigenic site whereas the lower pAb binds just below the 140-loop site.

In the same group of ferrets, we observed pAbs targeting two distinct NA epitopes: one on the lateral corner of NA and the other to the NA inter protomer interface (Fig. 5B). The first pAb, identified as a corner binder, has a similar angle of approach as FLUBV mAb-393 (*32*). We were only able to generate a three-dimensional (3D) reconstruction for the corner NA pAb and report a representative 2D class for the interface binding pAb.

Although these data reveal the targeting of pAbs to major antigenic sites of Victoria HA in the Victoria-infected ferrets, we wanted to assess whether any cross-reactive pAbs were generated from infection with a Yamagata strain. We complexed the sera from B/Oklahoma/10/2018-infected ferrets on day 28 with B/Washington/02/2019 HA and NA, which revealed no cross-reactive pAbs in line with the serology data (fig. S5D). Although ELISA data with rHA and rNA proteins showed some cross-reactivity, EMPEM analysis may not detect pAbs with high off-rates that can be lost during sample preparation and processing.

These data reveal major antigenic epitopes targeted by a contemporary Victoria infection against homologous HA and NA proteins. Furthermore, they confirm a lack of functional cross-reactive antibodies elicited from contemporary Yamagata infection toward the Victoria NA.

## DISCUSSION

This study aimed to understand the asymmetric immunity elicited by FLUBV lineage infections and to identify key immune mechanisms underlying this phenomenon. We established two animal models that replicate the observed cross-protection differences noted in limited human studies and found that this phenomenon is restricted to contemporary virus isolates, which display increased antigenic divergence. Using these models, we further found that functional NA-specific antibodies are the primary drivers of these protective immune differences.

Our findings indicate that, in ferrets, infection with the Yamagata lineage provides only limited cross-protection against Victoria lineage infections, whereas immunity induced by the Victoria lineage confers more robust cross-protection against Yamagata. Notably, preexisting H1N1 and Victoria immunity further enhanced cross-protection, whereas H1N1 and Yamagata immunity did not (Fig. 1, C to F). Although some ferrets from the H1N1/Victoria group exhibited measurable cross-reactive HI titers toward the Yamagata antigen day 14 postinfection, these titers declined by day 28 (fig. S1G). This suggests that the observed HI titers may have been generated by short-lived extrafollicular antibody responses and are unlikely to be the primary mediators of the enhanced protection.

We demonstrated that NA-specific antibodies elicited by contemporary Victoria lineage can mediate enhanced cross-lineage protection. However, it is likely that a combination of humoral and cellular factors contributes to the observed earlier viral clearance. Cross-reactive CD8+ T cells have been implicated in reducing disease severity and enhancing viral clearance for FLUBV lineages in humans (*33*, *34*). The >90% sequence homology between influenza B lineages suggests that, if cross-reactive CD8+ T cells contribute to protection, their effects should be similar for both lineages. This raises intriguing questions about why H1N1/Yamagata immunity fails to elicit comparable cross-reactive responses to those seen with H1N1/Victoria immunity.

H1N1 preimmunity alone did not enhance protection against primary FLUBV infection compared to naïve ferrets (fig. S1, E and F). This suggests that the enhanced cross-protection observed occurs specifically during secondary influenza exposure and is lineage dependent. This highlights a dynamic interaction between preexisting FLUAV and FLUBV immunity that selectively benefits Victoria lineage–mediated protection. Although cross-reactive neutralizing antibodies wane by day 28, nonneutralizing antibodies with effector functions, such as antibody-dependent cellular cytotoxicity, may play a more durable role in the enhanced cross-protection observed in the H1N1/Victoria group. These data identify a unique dynamic between FLUAV and FLUBV preimmunity and emphasize the need for further exploration of the mechanisms underlying these lineage-dependent effects.

We used the ferret model to assess not only differences in FLUBV cross-protection but also the durability of this protection. Victoria-induced immunity, especially with H1N1 preimmunity, remained durable and superior over a 6-month infection interval, whereas Yamagata-induced protection declined more rapidly. At 6 months, there was no difference in H1N1 and Yamagata preimmunity or Yamagata immunity alone compared to naïve animals on days 1 and 3 (Fig. 1F). The absence of functional cross-reactive antibodies following a recent Yamagata infection (fig. S1H) suggests that inadequate cell-mediated immune responses during the primary infection may contribute to the waning immunity observed with Yamagata viruses.

In alignment with our findings in ferrets, our mouse model demonstrates differential protection with contemporary viruses only, highlighting that this phenomenon does not extend to older clades. Primary infection with noncontemporary viruses provided cross-protection against not only other noncontemporary viruses but also contemporary strains. This could explain why reports of differential protection in humans have only emerged recently (*10*, *11*). Ancestral isolates may have offered more durable protection; however, the recent divergent evolution of contemporary viral isolates post-2015 appears to have disrupted this protection, particularly for Yamagata viruses. Although both lineages have shown increased endemic activity, they used distinct mechanisms for recent evolutionary success. Victoria viruses have evolved through traditional mechanisms, including major changes to the HA protein, whereas Yamagata viruses have faced stronger selective pressure on the NA protein (*7*, *35*). These differing evolutionary pressures, combined with limited reassortment events, have further diversified the lineages. In contrast, less contemporary viruses experienced frequent reassortment, such as in the early 2000s when the NA gene segment from Victoria lineage viruses was replaced by Yamagata lineage NA, introducing conserved epitopes that could elicit cross-reactive antibody responses to Yamagata viruses (*35*–*37*). The lack of reassortment and increased genetic diversity among contemporary viruses may explain the absence of observed cross-protection that was present with noncontemporary viruses.

Our study investigated the mechanisms behind differential FLUBV protection, demonstrating that cross-protection is antibody mediated. Passive transfer of immune serum from Victoria-infected animals showed cross-protective capabilities against Yamagata viruses, whereas serum from Yamagata infections provided only homologous protection. We found that HA antibodies elicited by contemporary FLUBVs are limited to homologous neutralizing responses but remain cross-reactive to nonneutralizing epitopes across a diverse set of evolutionarily distinct antigens (Fig. 3, A and B). Although stem-directed and nonneutralizing antibodies have been implicated in influenza cross-protection (*38*–*41*), our findings from vaccination experiments indicate that HA-specific immunity—whether neutralizing or not—is not responsible for FLUBV asymmetric cross-protection. Despite the diverse antibody responses observed in antigen arrays targeting multiple antigens spanning distinct clades and lineages, these antibodies ultimately confer only homologous protection.

For our experimental models, we deliberately chose infectious doses that allowed for notable viral replication in the respiratory tract of animals yet induced minimal clinical signs to elicit robust immune responses and enable subsequent reinfection studies. The noticeable decrease in viral replication and the more rapid viral clearance observed in animals with breakthrough infections when compared to the control animals prompted us to expand our studies to evaluate the influence of the NA-specific antibody responses on cross-protection against FLUBV. Although NA antibodies have been implicated in broad protection against heterologous strains of FLUAV and FLUBV (*42*), the role for infection-elicited cross-lineage protective NA antibodies has not been fully defined for FLUBV. NA-specific antibodies function by binding to the NA protein and blocking its enzymatic activity, preventing the virus from being released by infected cells and limits the ability of the virus to move within the respiratory tract (*43*). Hence, when NA-specific antibodies are involved in protection from infection, it can clinically result in reduced disease severity and duration of viral shedding, rather than complete viral neutralization, which is associated with HA-mediated protection as observed in both our mouse and ferret infection models. Furthermore, unlike HA, NA tends to undergo less antigenic variation (*44*), making it a promising target for inducing cross-protective immunity.

In our studies, NA-specific antibodies elicited from FLUBV infections demonstrated cross-reactivity across lineages and evolutionary clades, except in the case of contemporary Yamagata virus infections. Functional assessments using the ELLA in mice and ferrets, as well as vaccinations in mice, revealed that contemporary Yamagata anti-NA antibodies are unable to inhibit Victoria NA activity resulting in breakthrough infections. In addition, when we excluded all seven other gene segments and evaluated cross-protection elicited solely by rNA vaccination, we observed deficiencies in functional antibodies by ELLA, which corresponded to unsuccessful protection from a cross-lineage challenge. In contrast, vaccination with Victoria lineage rNA significantly reduced viral replication against both homologous and cross-lineage Yamagata challenges. Moreover, our EMPEM analysis confirms the absence of cross-reactive functional HA and NA pAbs induced by a Yamagata infection as none of the pAbs bound to Victoria HA or NA (Fig. 5C). Given the data presented in our studies, which suggest a deficiency in contemporary Yamagata-elicited antibodies for cross-protection against contemporary Victoria infections, these findings are expected. However, given the limited knowledge regarding the epitope-specific responses elicited by FLUBV infections in the ferret, it is crucial to continue investigating this response. Notably, our analysis also identified key regions targeted by Victoria pAbs following infection, including two head binders for HA and both an interface and corner binder for NA.

Before the disappearance of Yamagata viruses from human circulation, there had been increased genetic diversity, particularly in the HA and NA proteins, peaking in 2015 and 2018 (*7*). Our contemporary Yamagata virus isolate, B/Oklahoma/10/2018, represents a new clade 3A, characterized by NA substitutions I171M, D197N, and N342K (fig. S6). Previous studies have highlighted differences in NA enzymatic activity and FLUBV pathogenicity based on substitutions at position 342; however, their impact on subsequent heterologous cross-protection remains unclear (*45*, *46*). Contemporary Yamagata mutations may restrict functional NA-specific antibody responses to the homologous antigen, potentially allowing for breakthrough infections from Victoria lineage viruses, as the cross-reactive antibodies elicited by Yamagata infections are limited in functionality. If contemporary Victoria infections continue to induce cross-protection against both lineages, whereas Yamagata infections confer protection solely against Yamagata, a population with greater immunity to Yamagata viruses compared to Victoria viruses may emerge. These dynamics could help explain the dominance of Victoria lineage viruses prior to the emergence of SARS-CoV-2 (severe acute respiratory syndrome coronavirus 2) and the disappearance of the Yamagata lineage following the COVID-19 pandemic as fewer susceptible individuals remained. Future studies should focus on elucidating the molecular mechanisms underlying the NA protein mutations, such as I171M and N342K, and how these specific amino acid changes may influence cross-protection between FLUBV lineages.

In conclusion, this study provides insights into the mechanisms underlying differential cross-protection between contemporary influenza B virus lineages. Although current influenza vaccination strategies focus on the constantly evolving immunodominant head of HA, our findings highlight the superior role of NA-elicited immunity for FLUBV cross-lineage protection and identify critical regions for targeted immune responses. Given the recent absence of Yamagata detections, these insights could inform the design of next-generation influenza vaccines targeting NA aimed at potentially eliminating Victoria viruses from human circulation. Further research is needed to assess the long-term durability of FLUBV NA-mediated protection in both experimental and clinical settings.

## MATERIALS AND METHODS

### Virus propagation

The FLUBVs B/Washington/02/2019 [International Reagent Resource (IRR), FR-1709, E6], B/Oklahoma/10/2018 (IRR, FR-1660, C2/E1), B/Brisbane/60/2008 (St. Jude Children’s Research Hospital, E4), and B/Massachusetts/02/2012 (IRR, FR-1196, E6) were propagated in 9- to 11-day-old specific pathogen–free embryonated chicken eggs. Allantoic fluid was inoculated, and eggs were incubated at 35°C with 5% CO_2_ for 3 days. After embryo termination at 4°C overnight, allantoic fluid was collected and clarified by centrifugation at 4000*g* for 20 min and stored at −80°C until further use.

### Ferrets, mice, infections, and immunizations

Six- to 9-month-old, female ferrets (*Mustela putorius furo*) were obtained from Triple F farms and acclimated in the UGA animal facilities. Before arrival, ferrets underwent sterilization and scent gland removal. Animals were housed in pairs and had ad libitum access to food and water. Environmental enrichment was also provided. For infections, animals were anesthetized with isoflurane and intranasally inoculated with 10^6^ PFU of virus diluted in 1 ml (500 μl per nostril) of sterile phosphate-buffered saline (PBS). After infection, animals were monitored twice daily for clinical signs. Weights and temperatures were recorded every other day.

Six- to eight-week-old, female BALB/c mice were purchased from Charles River and used for all mouse experiments. For infections, mice were anesthetized using 5% isoflurane and intranasally inoculated with 30 μl of live virus at specified infectious doses. Mice were monitored daily for weight loss and clinical signs. Animals reaching institutionally defined humane end points were humanely euthanized in accordance with American Veterinary Association Guidelines. For immunizations, each mouse was intramuscularly injected with a 2- or 10-μg dose of rHA or rNA, respectively, diluted 1:1 with AddaVax (InvivoGen, catalog no. vac-adx-10) in the hind leg. All animal experiments were performed in accordance with protocols approved by the University of Georgia Institutional Animal Care Committee (IACUC) identification number D16-00276 (A3437-01).

### Sample collections

For ferrets, after each infection, nasal wash samples were collected on days 1, 3, 5, and 7 for analysis of viral replication. Although the animals were anesthetized, 3 ml of sterile PBS was flushed through their nostrils, collected, and stored at −80°C until further use. Whole blood was collected on day 0, prior to infection, and days 14 and 28 postinfection in a serum separator (Greiner Bio-One, ref. no. 454228P). The sample was centrifuged at 1700*g* for 7 min to separate out the serum for downstream analysis.

For mice, lung samples were collected into 1 ml of PBS at specified time points, mechanically homogenized, and clarified by centrifugation at 2500*g* for 2 min. Supernatant was collected, aliquoted, and stored at −80°C until quantification via plaque assay. Blood was collected in blood collection tubes (BD Microtainer, ref. no. 365967) at specified time points by submandibular or terminal bleeds and centrifuged at 10,000*g* for 2 min to separate serum. Serum was aliquoted and stored at 4°C until further use.

### Serum passive-transfer

Groups of 15 mice were inoculated with PBS or 10^3^ PFU of B/Washington/02/2019 or B/Oklahoma/10/2018 and terminally bled 35 days postinfection for serum collection and serological analysis. Serum was inactivated in a 56°C heat bath for 45 min. Individual animals’ serum was then assessed for antigen specificity using whole-virus ELISAs. Once reactivity was confirmed, serum from each group was pooled and 250 μl of inactivated serum was transferred by intraperitoneal injection into naïve recipient mice (*n* = 10 per group). Immediately following transfer, groups were challenged with 10^3^ PFU of either B/WA or B/OK. Lungs were collected 3 days postchallenge for assessment of viral replication.

### Influenza plaque assay

Madin-Darby Canine Kidney (MDCK-ATL; FR-926) cells were cultured in Dulbecco’s modified Eagle’s medium (DMEM; Corning, ref. no. 10-013-CV) supplemented with 5% fetal bovine serum (FBS) and 1x antibiotics-antimycotic (GenDEPOT, catalog no. 002-010) and incubated at 37°C with 5% CO_2_. To determine virus titers, MDCK cells were seeded with 4 × 10^5^ cells per well in a 12-well tissue culture treated plate (Thermo Fisher Scientific, ref. no. 130185) and incubated for 24 hours at 37°C with 5% CO_2_. Cells were then washed twice with 2x sterile PBS (Corning). Tenfold serial dilutions of virus stocks were prepared in DMEM with 0% FBS. MDCK cells were then inoculated with dilutions and incubated for 1 hour at 35°C with 5% CO_2_. Following incubation, the inoculum was overlaid with 0.8% avicel and MEM supplemented with 1 M Hepes, 200 mM l-glutamine, 7.5% NaHCO_3_, and 1X antibiotics-antimycotics and incubated at 35°C with 5% CO_2_ for 72 hours. Following incubation, overlay was decanted, and wells were washed twice with 2x PBS and then fixed with methanol:acetone (80:20). Subsequently, wells were stained with crystal violet, and plaques were counted to determine titers in plaque-forming units per milliliter (PFU/ml).

### Enzyme-linked immunosorbent assay

Ninety-six well microtiter plates (Greiner Bio-One, ref. no. 655001) were coated with 100 μl of a 1:100 dilution of either live B/WA or B/OK or recombinant protein at 1 μg/ml and incubated overnight at 4°C. Plates were then washed 3x with 0.05% PBS-T (PBS and 0.05% Tween 20) and blocked using 200 μl of 3% nonfat dry milk in 0.05% PBS-T for 2 hours at room temperature (RT). Serum was inactivated at 56°C for 45 min and serially diluted 10-fold in blocking buffer and then incubated for 2 hours at RT. Next, plates were washed 3x and horseradish peroxidase (HRP)–conjugated goat anti-mouse IgG secondary antibody (Thermo Fisher Scientific, catalog no. 31430) was diluted 1:3500 in blocking buffer and added to all wells (100 μl per well). Following a 1-hour incubation at RT, plates were washed 3x and 50 μl of 3,3',5,5'-Tetramethylbenzidine (TMB)-substrate solution (Vector Laboratories Inc. SKU-4400) was added to all wells for 10 min. The reaction was stopped with 50 μl of 0.5M H_2_SO_4_ and was read at a wavelength of 490 nm using Cytation 7 (BioTek).

### HI assay

To remove nonspecific inhibitors of HA, serum was treated with a 1:4 dilution of receptor-destroying enzyme (RDE) and incubated overnight (16 to 18 hours) at 37°C followed by a 30-min inactivation at 56°C and subsequent dilution to 1:10 with PBS. Before starting the assay, viral stocks were diluted to 8 hemagglutinating units (HAU) per 50 μl (4 HAU/25 μl). RDE-treated serum was added to a 96-well V-bottom plate, serially diluted twofold (starting from 1:2), and incubated for 30 min at RT after the addition of 25 μl of 4 HAU of virus. Subsequently, 50 μl of 0.5% turkey red blood cells was added and the plate was incubated for 30 min at RT. Following the final incubation, the plate was tilted, and results were recorded as the reciprocal of the highest dilution that prevented the virus from agglutinating.

### Enzyme-linked lectin assay

To determine viral NA activity, MaxiSorp surface 96-well microtiter plates were coated with fetuin (25 μg/ml; Sigma-Aldrich, catalog no. F3004-25MG) in coating buffer (PBS) and incubated at 4°C overnight. The following day, plates were washed 3x with PBS-T wash buffer (0.05% Tween 20). Virus was serially diluted twofold and 50 μl of viral dilutions along with 50 μl of sample diluent [PBS-Ca/Mg, 1% bovine serum albumin (BSA), and 0.5% Tween 20] were added to the plate and incubated at 37°C with 5% CO_2_ for 18 hours. After incubation, plates were washed 6x and 100 μl of peanut agglutinin–HRP (1:1000 dilution) was added to all wells for a 2-hour incubation at RT. Plates were washed 3x and developed with TMB-substrate solution (Vector Laboratories Inc. SKU-4400) for 15 min. The reaction was stopped with 100 μl of 0.5M H2SO4, and the optical density was read at 450 nm using Cytation 7 (Biotek).

To perform neuraminidase inhibition (NI) assays, plates were coated as described above. Heat-inactivated serum was diluted twofold in PBS at a starting dilution of 1:10. Fifty microliters of previously determined optimized virus dilution was added to 50 μl of serum dilutions. Fetuin-coated plates were washed 3x with PBS, and 100 μl of the virus:serum mixture was added to plates, which were incubated at 37°C for 18 hours. After incubation, plates were washed, developed, and read as described above.

### Plasmids, protein expression, and purification

Truncated rHA and rNA proteins from B/Washington/02/2019 and B/Oklahoma/10/2018 were cloned into pCAGGS or pTwist cytomegalovirus plasmid vectors. The plasmids were transformed into *Escherichia coli* (DH5α, NEB) and purified using E.Z.N.A. plasmid DNA maxiprep kits (Omega Bio-Tek) following the manufacturer’s protocol. Expression and purification of recombinant proteins were performed as previously described (*47*). In brief, a coculture of DNA, polyethyleneimine MAX (PEI MAX; Polysciences, catalog no. 24765-1), and Opti-MEM (Gibco, catalog no. 31985-070) were added to a suspension of EXPI293F cells and incubated for 5 to 7 days. Proteins were purified through a HisTrap excel column (Cytiva, 17371206) according to the manufacturer’s instructions. After purification, Western blotting and size exclusion chromatography (SEC) confirmed that the proteins had folded correctly.

### Spotting of influenza antigen microarray

*N*-Hydroxysuccinimide ester–derivatized hydrogel-coated slides (H slides type B, Schott-Nexterion, Germany) were spotted with rHA proteins of various influenza A and B strains, including the challenge strains B/Oklahoma/10/2018, B/Washington/02/2019, and A/Michigan/45/2015 (table S1). The microarray encompasses both FLUBV lineages, including six Victoria lineage strains and seven Yamagata lineage strains. rHA proteins of B/Oklahoma/10/2018, B/Washington/02/2019, and A/Michigan/45/2015 were spotted at four different dilutions with a concentration ranging from 130 to 16.25 μg/ml. All other proteins were spotted in a single concentration of 32.5 μg/ml. The proteins were diluted in sciSpot D1 spotting buffer (Scienion, Germany) with each dilution spotted in triplicate using a Scienion Sx noncontact array spotter. Sixteen identical microarrays were spotted on each 25 mm–by–75 mm slide.

### Profiling antibodies using influenza antigen microarrays

Mouse serum samples were diluted in incubation buffer (1% BSA and 0.025% Tween 20). Serum samples were diluted 1:1600 for IgG profiling or 1:25 for IgA profiling. The microarrays were blocked by 1-hour incubation on a rocker at RT with a chemical blocking solution [50 mM ethanolamine and 50 mM borate (pH 9.0)], washed twice with PBST (containing 0.05% Tween), twice with PBS, and once with deionized water (DDW) (each wash, 3 min on the rocker), and dried by centrifugation at RT for 5 min at 800*g*. The diluted samples were then incubated on preblocked microarrays for 2 hours at RT, in divided trays on the rocker. Slides were then washed twice with PBST and twice with PBS, and bound antibodies were detected by incubation for 45 min with fluorescently labeled secondary anti-mouse IgG or IgA antibodies diluted in incubation buffer (IgG: Alexa Fluor 647–conjugated AffiniPure, goat anti-mouse IgG, FCγ fragment specific, 115-605-008, Jackson ImmunoResearch, dilution: 1:1000; IgA: goat anti-mouse IgA-AF647, 1040-31, SouthernBiotech, dilution: 1:500). Arrays were then washed by PBST, PBS, and DDW and dried by centrifugation as described above. Slides were scanned using an Innoscan 1100 AL laser scanner (Innopsys Inc., USA), and the images were annotated with GenePix Pro version 7 software. Local background was subtracted from the mean fluorescence intensity of each spot, and the median fluorescence intensity was calculated for each triplicate of spots. Negative control arrays were incubated with incubation buffer only, and their MFI results were subtracted from all other arrays. Following annotation, area under the curve was computed to quantify the antibody responses for the antigens that were spotted at multiple dilutions. The data were then analyzed using in-house Python scripts.

### Electron microscopy polyclonal epitope mapping

EMPEM was performed as described previously (*48*) with the following modifications. Protein A beads were used instead of Capture Select during serum processing. For complexing, 0.185 μg for Oklahoma and 0.431 μg for Washington were complexed with 10 μg of HA and 15 μg of NA simultaneously and incubated at RT for 20 hours. Immune complexes were purified by SEC using a HiLoad 16/600 Superdex pg200 (GE Healthcare) column, with tris-buffered saline as the running buffer. SEC fractions corresponding to the complex were pooled and concentrated for staining. Samples were imaged on at Talos F200c operating at −200 kV and ×73,000 magnification using a Ceta 16M detector at 2 Å/pixel. Images were processed using RELION-4 PMID: 34783343. Because both HA and NA antigens were present in the samples, following the first round of reference-free 2D classification, two sample selections were made—one for HA and the other for NA. These two substacks underwent a second round of 2D classification after which only particles with pAbs bound were selected for 3D classification. From this point, EMPEM remains the same as previously described, with the final analysis performed in Chimera (*49*).

### Statistical analysis

Data were analyzed using appropriate statistical tests based on the experimental design. For comparisons involving multiple groups, analysis of variance (ANOVA) was performed to assess significant differences between group means. Post hoc tests were applied when necessary to identify specific group differences. Each animal is represented by an individual data point. All data are presented as means ± SD, and statistical significance was defined as *P* < 0.05. Statistical analyses were conducted using GraphPad Prism, and details of the statistical test used for each comparison and *P* values are given in the figure legends.
