# Supplementary material for: Neuraminidase-specific antibodies drive differential cross-protection between contemporary FLUBV lineages
Source: Sci Adv. 2025 Mar 28;11(13):eadu3344. doi: 10.1126/sciadv.adu3344 (PMC11952091; doi:10.1126/sciadv.adu3344)
Supplement: Supplementary file 1 — Figs. S1 to S6 Table S1 [file sciadv.adu3344_sm.pdf]

Science Advances
AAAS

Supplementary Materials for

Neuraminidase-specific antibodies drive differential cross-protection between contemporary FLUBV lineages

Caroline K. Page *et al.*

Corresponding author: Stephen M. Tompkins, smt@uga.edu

Sci. Adv. **11**, eadu3344 (2025)
DOI: 10.1126/sciadv.adu3344

This PDF file includes:

Figs. S1 to S6
Table S1

Supplemental Figures

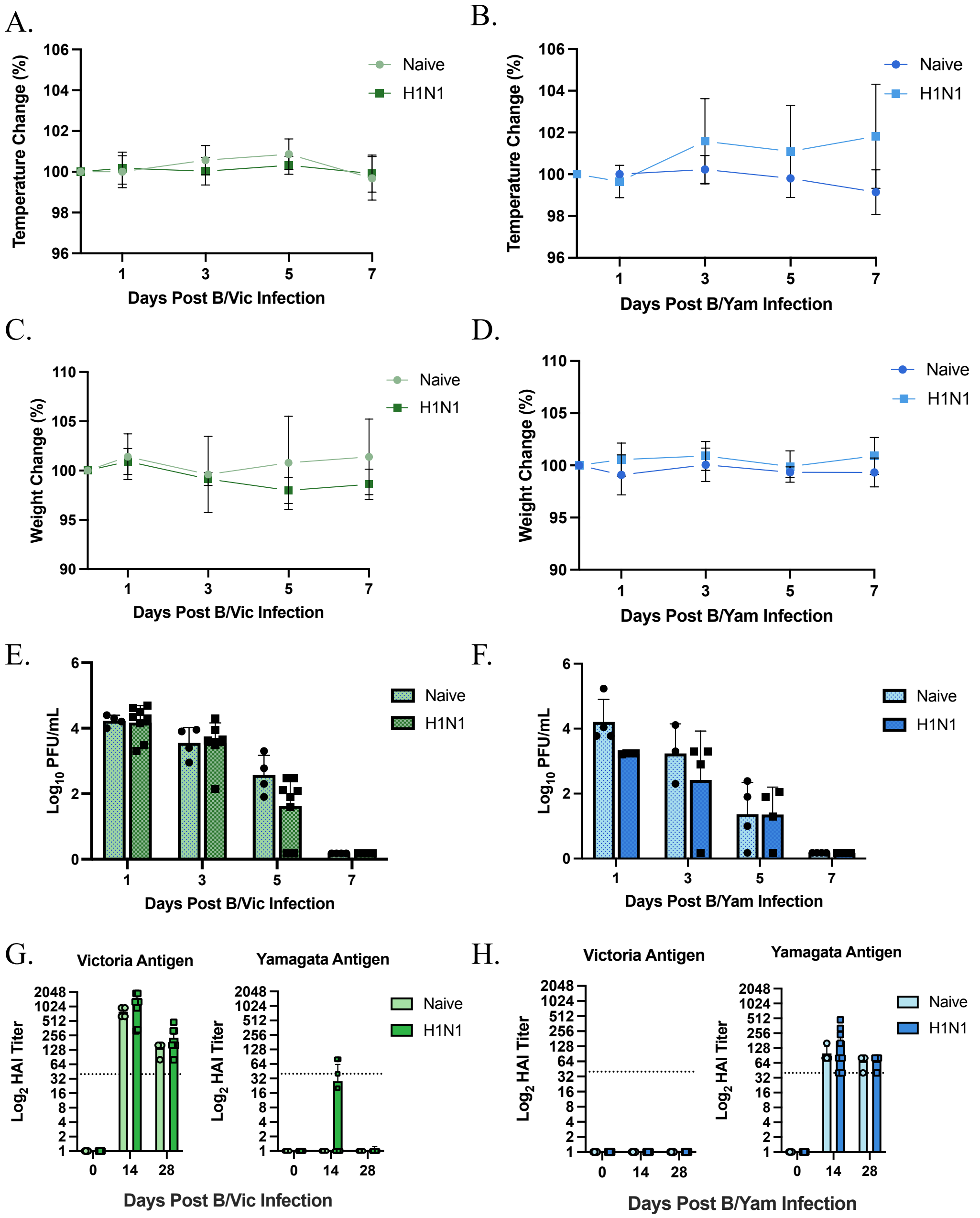

Figure S1. Clinical signs and viral replication following initial FLUBV infection in ferrets. (A-B) Temperature change and (C-D) weight loss in naïve or A/California/07/2009 (H1N1) pre-immune ferrets following intranasal inoculation with 10^6 PFU of contemporary B/Washington/02/2019 (B/Vic) or B/Oklahoma/10/2018 (B/Yam). (E-F) Viral replication in the upper respiratory tract measured from nasal washes collected on days, 1, 3, 5 and 7 post-infection with B/Vic or B/Yam virus. (G-H) HI titers in ferrets prior to infection and on days 14 and 28 post-infection with contemporary FLUBVs. Green bars indicate a B/Vic infection, and blue bars indicate a B/Yam infection. Statistical significance was determined using a one-way ANOVA, with $p < 0.05$ (*), $p < 0.01$ (**), $p < 0.001$ (***), and $p < 0.0001$ (****).

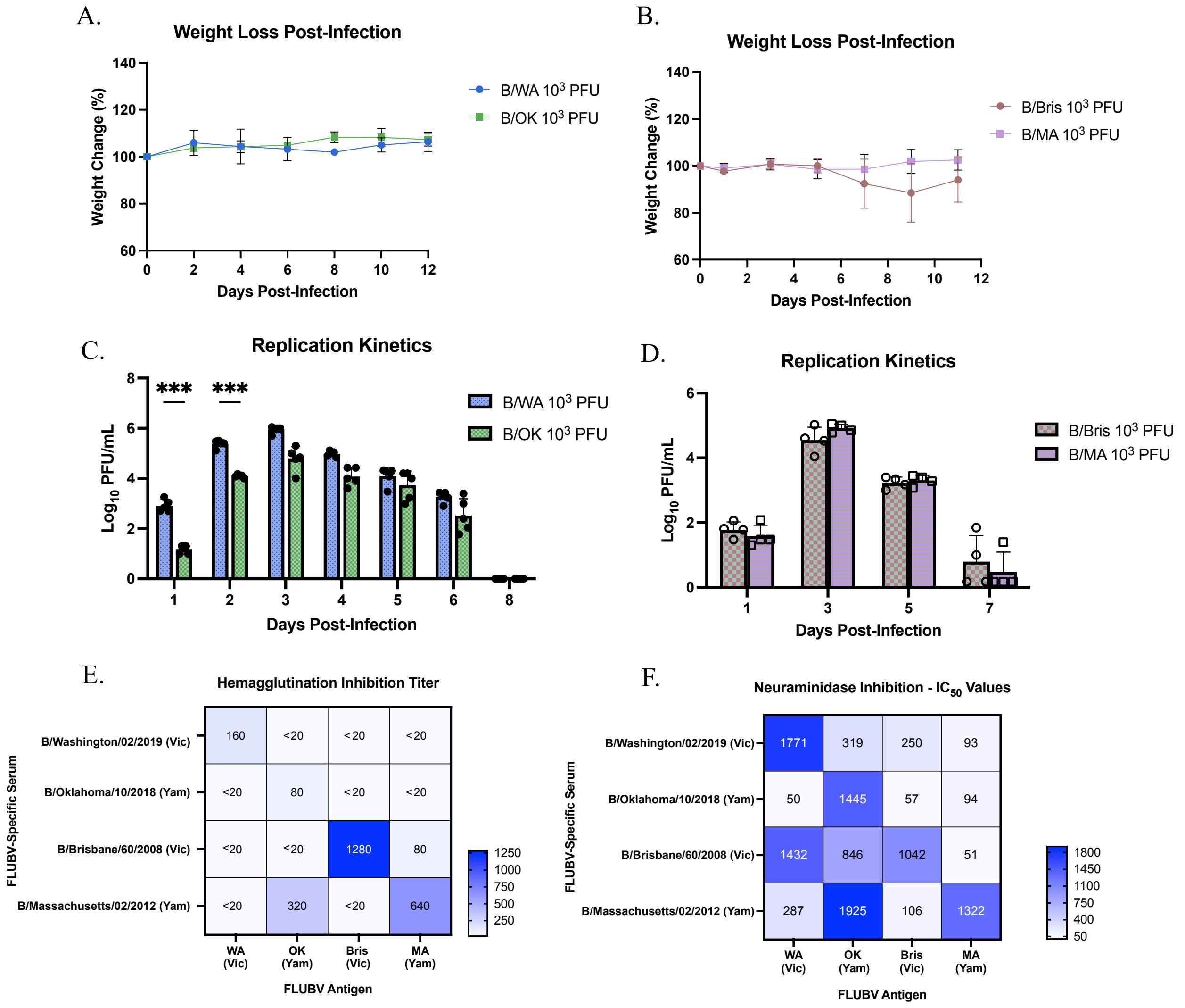

Figure S2. Weight loss and viral replication following FLUBV infection in mice (A-B) Weight loss in mice following intranasal inoculation with 10^3 PFU B/Washington/02/2019 (B/WA), B/Oklahoma/10/2018 (B/OK), B/Brisbane/60/2008 (B/Bris), or B/Massachusetts/02/2012 (B/MA). (C-D) Viral replication in the lungs of mice following FLUBV infection. (E) The heatmaps display hemagglutination inhibition (HAI) titers and (F) IC50 values calculated from ELLA inhibition curves for various FLUBV antigens. Each column represents a distinct antigen, and each row corresponds to serum samples collected from mice 35 days post-infection with B/Washington/02/2019 (Vic), B/Oklahoma/10/2018 (Yam), B/Brisbane/60/2008 (Vic), or B/Massachusetts/02/2012 (Yam). Darker shades indicate higher values, reflecting stronger inhibition, while lighter shades represent lower values, indicative of weak or no inhibition. Data are averaged from triplicate measurements, statistical significance was determined using a two-way ANOVA, with $p < 0.05$ (*), $p < 0.01$ (**), $p < 0.001$ (***), and $p < 0.0001$ (****).

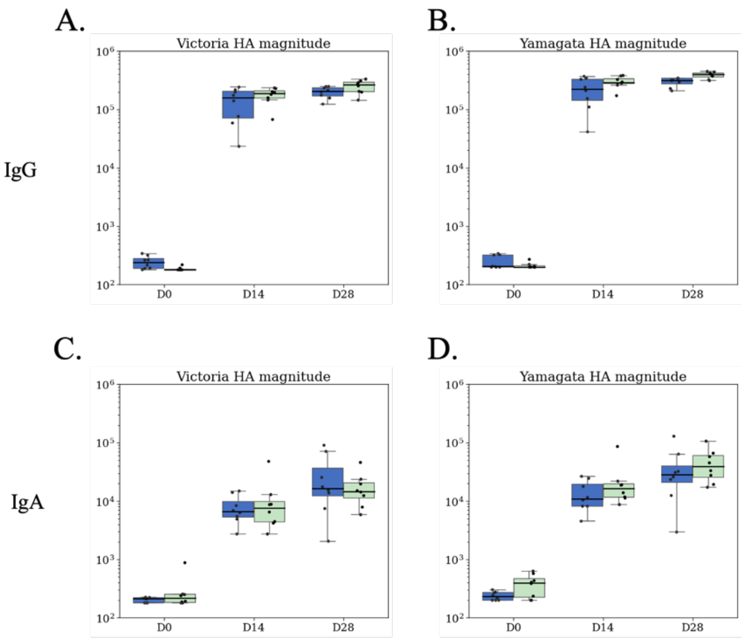

Figure S3. Cumulative response to microarray antigens following contemporary FLUBV infection. Median box plots, plotted on a logarithmic scale, represent the magnitude of (A-B) IgG and (C-D) IgA serum responses elicited by B/Washington/02/2019 (blue) and B/Oklahoma/10/2018 (green) infections in mice on days 0 (D0), 14 (D14), and 28 (D28) post-infection, to a diverse set of FLUBV antigens (Table S1). (A) and (C) represent responses to only the Victoria lineage antigens (n=6) included in the microarray, and (B) and (D) represent responses to Yamagata lineage antigens (n=7). The magnitude was calculated as the sum of antibody binding to Victoria or Yamagata HA proteins, as measured by background-subtracted median fluorescence intensity (MFI).

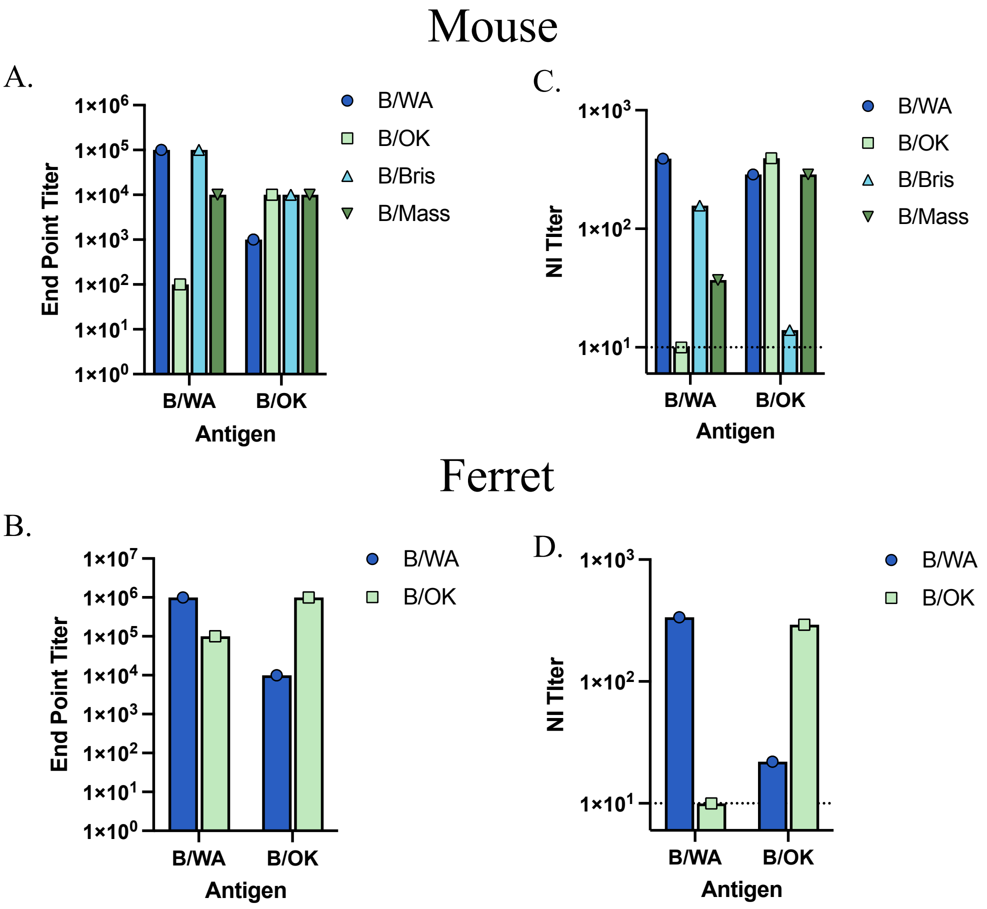

Figure S4. Summary of neuraminidase response to FLUBV infection in mice and ferrets. Summary data from Figure 4 showing endpoint titers measured by (A-B) ELISA and (C-D) ELLA assays. (A-C) Serum collected from mice infected with B/Washington/02/2019 (B/WA), B/Oklahoma/10/2018 (B/OK), B/Brisbane/60/2008 (B/Bris), or B/Massachusetts/02/2012 (B/Mass) were tested for reactivity against the B/WA and B/OK antigen (x-axis). (B-D) Serum collected from ferrets infected with B/Washington/02/2019 (B/WA) or B/Oklahoma/10/2018 (B/OK) tested against the B/WA and B/OK antigen (x-axis). End point titers for the ELISA were determined as the highest dilution giving a signal above the threshold. Neuraminidase inhibition (NI) titers were defined as the reciprocal dilution achieving 50% inhibition of NA activity. The dashed line represents the limit of detection for the assay.

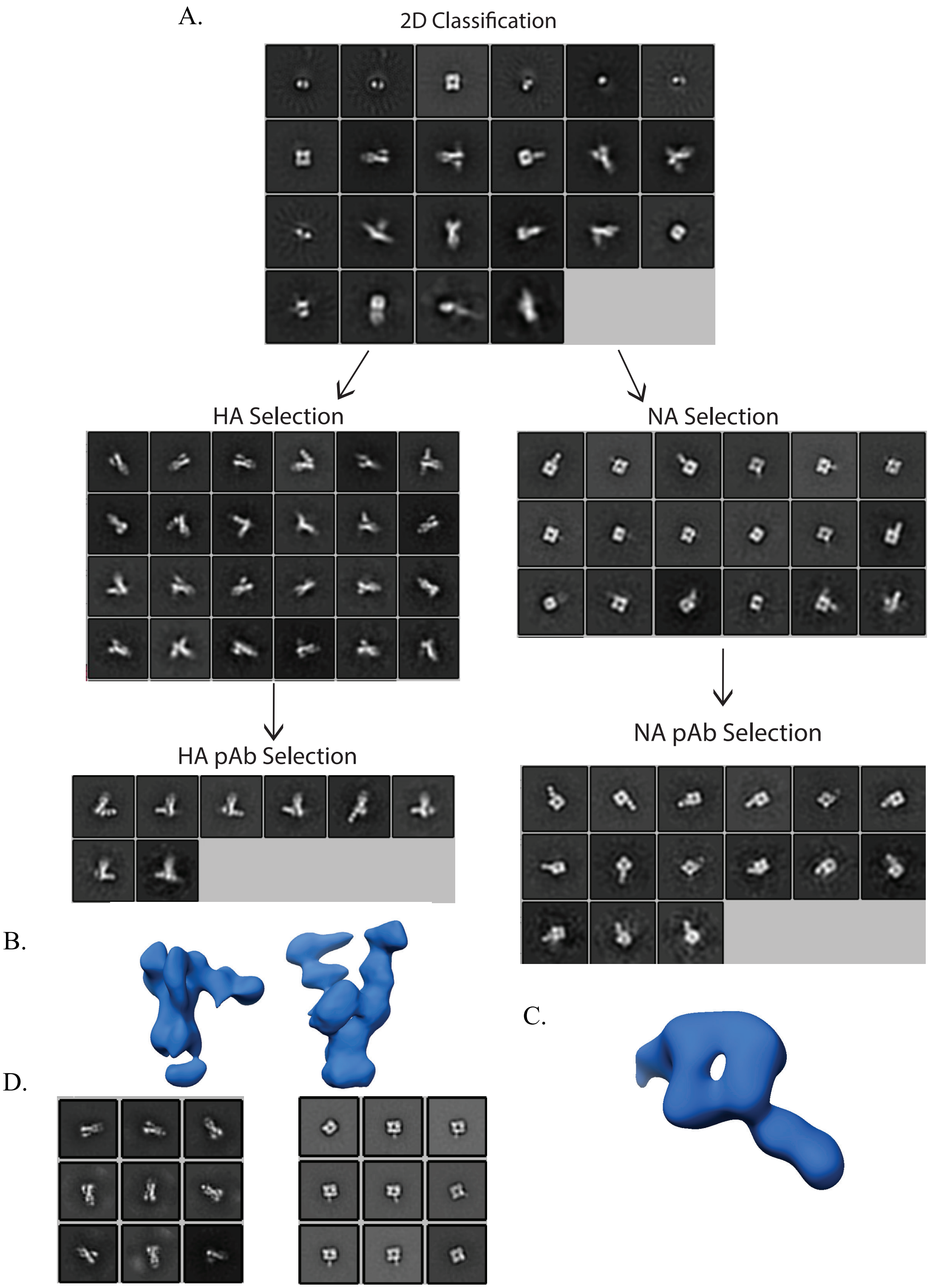

Figure S5. Workflow for separating HA and NA particles and analyzing pAb responses. (A) Workflow for separating HA and NA particles in the same dataset. Initial selections were made for classes containing either HA or NA particles. After a subsequent round of 2D classification, classes containing pAbs were selected and underwent 3D classification. (B) 3D classes for pAbs used for the pAb models in Figure 5A. (C) 3D class used for the pAb model in Figure 5B. (D) 2D classes for Oklahoma ferrets, showing the absence of pAb responses for these antigens.

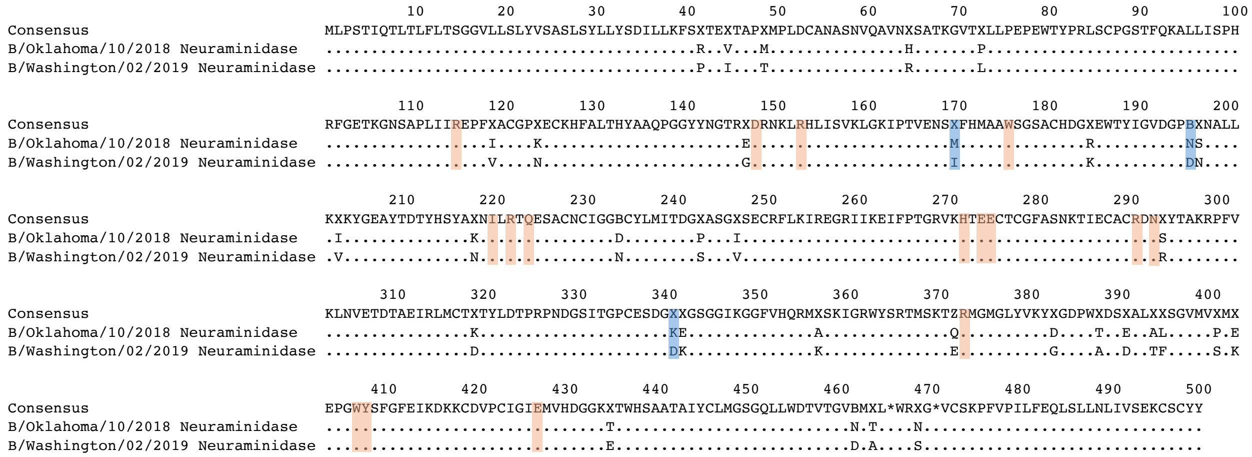

Figure S6. Amino acid sequence alignment of FLUBV neuraminidase proteins. Amino acid sequences of neuraminidase (NA) proteins from contemporary influenza B viruses (FLUBV) of the Victoria lineage (B/Washington/02/2019) and Yamagata lineage (B/Oklahoma/10/2018) were aligned. Conserved residues comprising the active site are shaded in red, while residues highlighted in blue indicate known mutations specific to the Yamagata clade 3A. Sequence alignment was performed using Geneious software. This alignment underscores both conserved features critical to NA function and lineage-specific variations that may influence antigenicity and enzymatic activity.

Table S1. Antigens included in the microarray. The list is organized by subtype, corresponding number in the spider plot, strain name, and the year the strain was included in the vaccine (if applicable). This table is associated with spider plots in Figure 3A.

Subtype	Numbers in spider plots	Strain	Protein	Vaccine year (northern hemisphere)	Source	Cat#
Yamagata	88	B/Yamagata/16/1988	rHA1	-	Sino	40157-V08H1
	06a	B/Florida/4/2006	rHA	2008-2009	Sino	11053-V08H
	06b	B/Florida/4/2006	rHA	2008-2009	BEI	NR-15169
	12	B/Utah/02/2012	rHA1	-	Sino	40463-V08H1
	13a	B/Phuket/3073/2013	rHA	2015-2016, 2017-2022	Sino	40498-V08B
	13b	B/PHUKET/3073/2013	rHA1	2015-2016, 2017-2022	Sino	40498-V08H1
	18	B/Oklahoma/10/2018*	rHA	-	In-House	
Victoria	04	B/Malaysia/2506/2004	rHA	2006-2008	Sino	11716-V08H
	08	B/Brisbane/60/2008	rHA	2010-2017	Sino	40016-V08H
	17	B/Colorado/06/2017	rHA	2018-2020	Native Antigens	Custom: ARQ85589
	19a	B/Washington/02/2019	rHA	2020-2022	Sino	40722-V08H
	19b	B/Washington/02/2019*	rHA	2020-2022	In-House	
	21	B/Austria/1359417/2021	rHA	2022-2024	Sino	40862-V08H
H3N2	68	A/Aichi/2/1968	rHA	-	Sino	11707-V08H
	89	A/Guizhou/54/1989	rHA1	-	Sino	40480-V08H1
	97	A/Sydney/5/1997	rHA	1998-2000	Sino	40149-V08B
	02	A/Fujian/411/2002	rHA	2004-2005	Sino	40120-V08B
	04a	A/NewYork/55/2004	rHA1	-	Sino	40436-V08H1
	04b	A/California/7/2004	rHA1	2005-2006	Sino	40118-V08H1
	04c	A/California/7/2004	rHA	2005-2006	Sino	40118-V08B
	05	A/Wisconsin/67/2005	rHA	2006-2008	Sino	11972-V08H
	07	A/Brisbane/10/2007	rHA	2008-2010	Sino	11056-V08H
	09a	A/Perth/16/2009	rHA	2010-2012	Sino	40043-V08H
	09b	A/Victoria/210/2009	rHA	2010-2012	Sino	40058-V08B
	11	A/Victoria/361/2011	rHA1	2012-2013	Sino	40145-V08H1
	12	A/Texas/50/2012	rHA1	2013-2015	Sino	40354-V08H1
	13	A/Switzerland/9715293/2013	rHA	2015-2016	Sino	40497-V08B
	14a	A/HongKong/4801/2014	rHA1	2016-2018	Sino	40555-V08H
	14b	A/Hong Kong/4801/2014	rHA2	2016-2018	eEnzyme	IA-HA2-714P
	16	A/Singapore/INFIMH-16-0019/2016	rHA	2018-2019	Native Antigens	Custom: EPI1140322
	17	A/Kansas/14/2017	rHA	2019-2020	Native Antigens	Custom: AVG71503.1
	19a	A/HongKong/2671/2019	rHA	2020-2021	Sino	40721-V08H
	19b	A/HongKong/45/2019	rHA	2020-2021	Sino	40765-V08H
	20	A/Cambodia/e0826360/2020	rHA	2021-2022	Sino	40789-V08H
	21	A/Darwin/6/2021	rHA	2022-2024	Sino	40868-V08H
H1N1	33	A/WSN/1933	rHA	-	Sino	11692-V08H
	34a	A/PuertoRico/8/1934	rHA	-	Native Antigens	FLU-H1N1-HA-100
	34b	A/PuertoRico/8/1934	rHA	-	Sino	11684-V08H
	77	A/USSR/90/1977	rHA1	-	Sino	40134-V08H1
	95	A/Beijing/262/1995	rHA1	1998-2000	Sino	40133-V08H1
	99	A/NewCaledonia/20/1999	rHA	2000-2007	Sino	11683-V08H1
	06	A/SolomonIslands/3/2006	rHA	2007-2008	Sino	11708-V08H
	07	A/Brisbane/59/2007	rHA	2008-2010	Sino	11052-V08H
	09a	A/California/07/2009	rHA	2010-2017	Sino	11085-V08H
	09b	A/California/07/2009	rHA	2010-2017	Native Antigens	Custom: ACP44189.1
	15a	A/Michigan/45/2015	rHA1	2017-2019	Sino	40567-H08H
	15b	A/Michigan/45/2015	rHA	2017-2019	Sino	40567-V08H1
	15c	A/Michigan/45/2015*	rHA	2017-2019	In-House	
	18a	A/Brisbane/02/2018	rHA	2019-2020	Native Antigens	Custom: EPI1440504
	18b	A/Brisbane/02/2018	rHA	2019-2020	Sino	40719-V08H
	19a	A/Wisconsin/588/2019	rHA	2021-2023	Sino	40787-V08H1
	19b	A/Hawaii/70/2019	rHA	2020-2021	Sino	40717-V08H

* Spotted in serial concentrations and AUC was calculated
